# Identification and Expression Analysis of Hexokinases Family in *Saccharum spontaneum* L. under Drought and Cold Stresses

**DOI:** 10.3390/plants12061215

**Published:** 2023-03-07

**Authors:** Ying Liu, Yaolan Jiang, Xiaolan Liu, Hefen Cheng, Yuekun Han, Dawei Zhang, Jinfeng Wu, Lili Liu, Mingli Yan, Youxiong Que, Dinggang Zhou

**Affiliations:** 1 Hunan Key Laboratory of Economic Crops Genetic Improvement and Integrated Utilization, School of Life and Health Sciences, Hunan University of Science and Technology, Xiangtan 411201, China; 2 Crop Research Institute, Hunan Academy of Agricultural Sciences, Changsha 410000, China; 3 Key Laboratory of Sugarcane Biology and Genetic Breeding, Ministry of Agriculture/National Engineering Research Center for Sugarcane, Ministry of Science and Technology, Fujian Agriculture and Forestry University, Fuzhou 350002, China; 4 Key Laboratory of Ecological Remediation and Safe Utilization of Heavy Metal-Polluted Soils, College of Hunan Province, Xiangtan 411201, China

**Keywords:** *Saccharum spontaneum*, hexokinase, genome-wide analysis, transcriptome, expression patterns

## Abstract

In plants, the multi-gene family of dual-function hexokinases (HXKs) plays an important role in sugar metabolism and sensing, that affects growth and stress adaptation. Sugarcane is an important sucrose crop and biofuel crop. However, little is known about the *HXK* gene family in sugarcane. A comprehensive survey of sugarcane *HXKs*, including physicochemical properties, chromosomal distribution, conserved motifs, and gene structure was conducted, identifying 20 members of the *SsHXK* gene family that were located on seven of the 32 *Saccharum spontaneum* L. chromosomes. Phylogenetic analysis showed that the *SsHXK* family could be divided into three subfamilies (group I, II and III). Motifs and gene structure were related to the classification of *SsHXKs*. Most *SsHXKs* contained 8–11 introns which was consistent with other monocots. Duplication event analysis indicated that *HXKs* in *S. spontaneum* L. primarily originated from segmental duplication. We also identified putative cis-elements in the *SsHXK* promoter regions which were involved in phytohormone, light and abiotic stress responses (drought, cold et al.). During normal growth and development, 17 *SsHXKs* were constitutively expressed in all ten tissues. Among them, *SsHXK2*, *SsHXK12* and *SsHXK14* had similar expression patterns and were more highly expressed than other genes at all times. The RNA-seq analysis showed that 14/20 *SsHXKs* had the highest expression level after cold stress for 6 h, especially *SsHXK15*, *SsHXK16* and *SsHXK18*. As for drought treatment, 7/20 *SsHXKs* had the highest expression level after drought stress for 10 days, 3/20 (*SsHKX1*, *SsHKX10* and *SsHKX11*) had the highest expression level after 10 days of recovery. Overall, our results revealed the potential biological function of *SsHXKs*, which may provide information for in-depth functional verification.

## 1. Introduction

Sugars are not only major components of cellular architecture, but also the main energy sources of life activities [1]. In higher plants, sugars regulate plant growth and development by being involved in many vital processes, including glycolysis, tricarboxylic acid cycle, photosynthesis, growth, and stress responses [2,3]. Sugars can affect gene expression in plants not only through general metabolic effects, but also as signal molecules by interacting with sensor proteins [2]. Hexokinases (HXKs, EC 2.7.1.1) are the most important glucose signaling proteins and in higher plants, and are the essential enzymes in carbohydrate metabolism, which can regulate glycolytic flux by controlling the entry point of glucose into glycolysis [4,5,6,7]. HXKs also play important regulatory roles as the major enzymes in sugar metabolism and sugar signaling in plants through catalyzing irreversible reactions, such as the first irreversible catalytic enzyme in the glycolytic pathway [5,6]. In addition, HXKs can provide substrates for various pathways, such as starch synthesis and fatty acid synthesis [4,5,8].

Generally, hexokinases with both signal transduction and hexose phosphorylation functions are called HXK, while hexokinases with only glycol signal transduction functions are called hexokinase-like (HKL) [9]. In *Arabidopsis thaliana*, among the identified six AtHXKs proteins, three of them (AtHXK1, AtHXK2 and AtHXK3) can phosphorylate hexoses, while the other three (AtHKL1, AtHKL2 and AtHKL3) lack the ability of hexose phosphorylation and only have signal transduction functions [10]. AtHXK1 is the most representative glucose sensor involved in plant development and stress response [7,11]. Lugassi et al. found that overexpression of *AtHXK1* in citrus was able to cause guard cells generated stomatal closure, which was consistent with the findings that increased expression of HXK in guard cells could accelerate stomatal closure by Kelly et al. [12]. Dai et al., showed that overexpression of *AtHXK1* in *Solanum lycopersicum* L. led to reduced photosynthesis, slow growth and the induction of senescence, which indicated that *AtHXK1* also plays an important role in photosynthetic tissues [13]. In *Malus × domestica*, Hu et al. found that *MdHXK1* could regulate anthocyanin biosynthesis by phosphorylating MdbHLH3 [14], and Sun et al. found that MdHXK1 can also promote Na^+^ accumulation in the vacuole and improve salt tolerance at least partially in a MdNHX1-dependent manner [15]. Li et al. found that suppression of *SlHXK1* led to accelerated leaf senescence and stunted plant growth and that downregulation of *SlHXK1* caused more starch to accumulate in tomato leaves [16]. However, the functions of HKL proteins are poorly understood. Due to its lack of glucose-phosphorylation activity, AtHKL1 proteins usually function as a negative regulator of plant growth [2].

Environmental stresses, especially those affecting water availability and temperature, such as drought and cold, are the major limitations of plant growth and development [17]. To survive these stresses, plants have developed sophisticated mechanisms, both on an external level (perception) and on an internal level (reaction) [18]. Non-enzymatic antioxidants such as glutathione are the first-line compounds against biotic/abiotic stress, which could be classified as the external perception level [19,20]. HXKs are involved in stress adaptation and defense regulation in plants as the internal level. Wang et al. reported that some *Jatropha curcas HXK* genes (*JcHXK1*, *JcHXK2*, *JcHXK3* and *JcHXK4*) were up-regulated in the leaves after cold stress and *JcHXK3* remarkably demonstrated cold-induced expression in the roots [21]. Han et al. discovered that HXK protein could significantly reduce the resistance to *Monilinia fructicola* infection in peach fruit [22]. Lugassi et al. found that the *AtHXK1* transgenic tobacco can suffer salt and drought stresses due to lower transpiration rates [23]. Küstner et al. compared the *gin2-1* mutant(Δ*HXK1*) with its wildtype, and revealed that the mutant plants responded more slowly to highlight stress [24].

Sugarcane (*Saccharum* spp. hybrid) is a C4 Poaceae grass, which is widely grown in tropical and subtropical areas, due to its high yield of sugar [25,26]. Sugarcane is also one of the most important industrial crops, which accounts for approximately 80% of sucrose and 40% of the ethanol global production around the world [26]. The HXK activity is positively correlated with the sugar content and the sugar content significantly influences the sugar crop’s and fruit’s quality [1]. HXKs are functionally important and have been widely reported in many plant species; however, the sugarcane *HXK* gene family members have not yet been identified and characterized, and the functions of HXK-related features in sugarcane are still poorly understood. In the present study, a comprehensive survey of *HXK* encoding genes in *S. spontaneum* L. based on genome-wide analysis was conducted and the expression analysis of *HXK* genes was also performed using RNA-seq data and SRA data. Overall, our results revealed the potential biological function of *SsHXKs*, which may provide information for in depth functional verification.

## 2. Results

### 2.1. Identification, Physicochemical Properties and Chromosomal Distribution of SsHXK Genes

**Figure 1:**
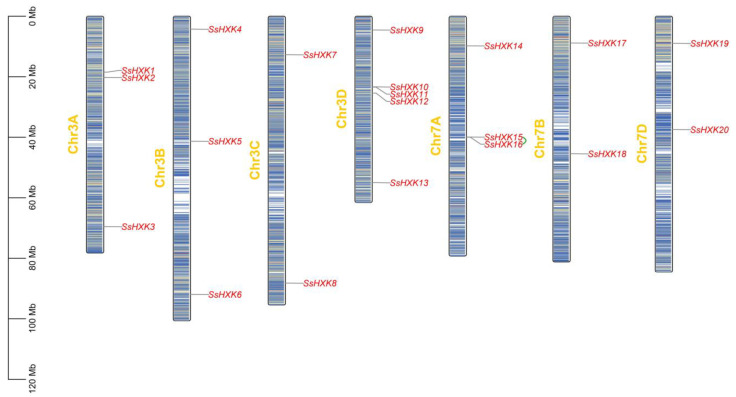
The chromosomal distribution of *SsHXKs*. The green line between the two gene names represented that they were tandem gene pair. Scale bar on the left indicates the chromosome lengths (Mb).

In total, we identified 20 *HXK* genes from the *S. spontaneum* genome using HMMER software and blast program. The amino acid lengths ranged from 211 aa (*SsHXK20*) to 654 aa (*SsHXK14*), the molecular weight ranged from 23,062.57 Da (*SsHXK20*) to 70,491.44 Da (*SsHXK14*), and their isoelectric points ranged from 4.96 (*SsHXK18*) to 9.45 (*SsHXK14*) (Table S1). In addition, *S. spontaneum HXK* genes prediction was localized in the chloroplast (ten *SsHXKs*), cytoplasmic (eight *SsHXKs*), mitochondrial (*SsHXK8*) and plasmalemma (*SsHXK2*) (Table S1). Based on their order on the chromosome, the *SsHXK* genes were named from *SsHXK1* to *SsHXK20*. The chromosome distribution of *SsHXKs* was uneven, and only seven out of 32 chromosomes were distributed (Chr3A, Chr3B, Chr3C, Chr3D, Chr7A, Chr7B and Chr7D) (Figure 1). Moreover, one tandem repeat gene pair (*SsHXK15*/*SsHXK16*) was identified.

### 2.2. Phylogenetic Analysis and Synteny Analysis

**Figure 2:**
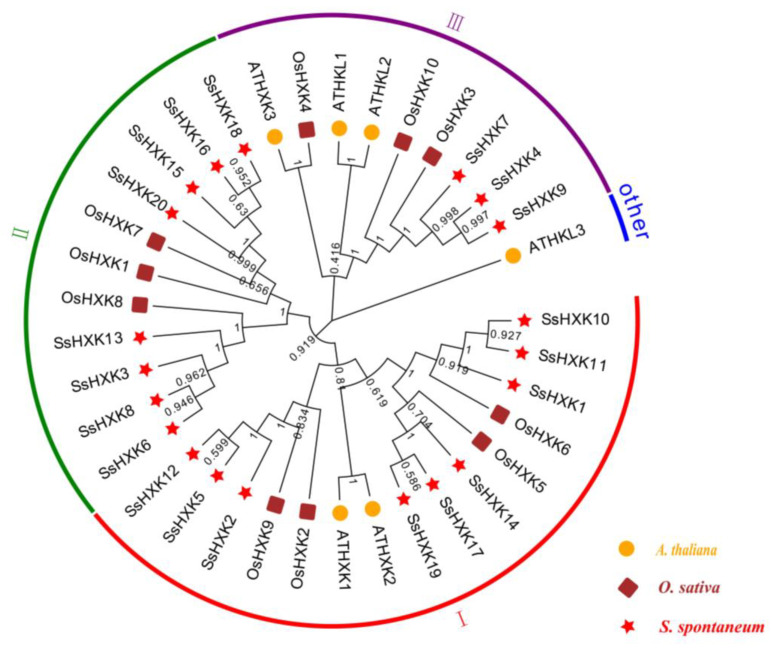
Phylogenetic analysis of the SsHXK proteins. The neighbor-joining tree (N-J tree, 1000 boosted replicates) were constructed using MEGA X. Proteins from AP85-441(*S. spontaneum*), rice (*O. sativa*) and *A. thaliana* are represented by stars, squares, and circles, respectively.

To investigate the evolutionary relationships of the SsHXK proteins, an unrooted neighbor-joining phylogenetic tree were conducted based on complete protein sequences among *S. spontaneum*, *Oryza sativa* and *A*. *thalian* (Figure 2). According to the phylogenetic tree of SsHXKs from three species, all SsHXK protein sequences were divided into four groups, named group I, group II, group III and other. Nine SsHXK proteins (SsHXK1, SsHXK2, SsHXK5, SsHXK10, SsHXK11, SsHXK12, SsHXK14, SsHXK17 and SsHXK19), four OsHXK proteins (OsHXK2, OsHXK5, OsHXK6 and OsHXK9) and two AtHXK proteins (AtHXK1 and AtHXK2) were classed into group I. Three SsHXK proteins (SsHXK4, SsHXK7, and SsHXK9) and three OsHXK proteins (OsHXK3, OsHXK4 and OsHXK10), two AtHKL proteins (AtHKL1 and AtHKL2) and one AtHXK protein (AtHXK3) were classed into group III. However, eight SsHXK proteins (SsHXK3, SsHXK6, SsHXK8, SsHXK13, SsHXK15, SsHXK16, SsHXK18 and SsHXK20) and three OsHXK proteins (OsHXK1, OsHXK7 and OsHXK8) were classed into group II, in which neither AtHXK protein no AtHKL protein was included. Even then, only one member, AtHKL3 independently forms a branch, which named other.

**Figure 3:**
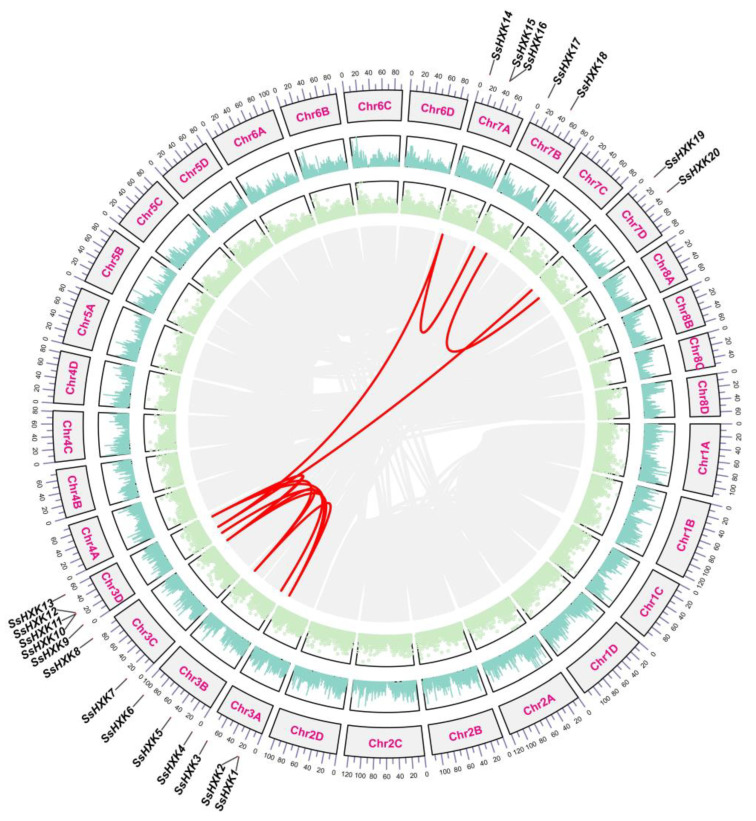
Schematic representations highlighting the interchromosomal relationships of the *SsHXK* genes. Gray lines indicate all syntenic blocks in the sugarcane genome, and the red lines indicate duplicated *HXK* gene pairs.

Segmental or tandem duplicates in many gene families are the main expanding pattern in plants. We predicted the *SsHXK* genes duplication events by the MCScanX (Figure 3). Eleven *HXK* gene pairs were identified as fragment duplicates (*SsHXK3*/*SsHXK6*, *SsHXK3*/*SsHXK8*, *SsHXK3*/*SsHXK13*, *SsHXK4*/*SsHXK9*, *SsHXK6*/*SsHXK8*, *SsHXK6*/*SsHXK13*, *SsHXK8*/*SsHXK13*, *SsHXK10*/*SsHXK14*, *SsHXK11*/*SsHXK19*, *SsHXK14*/*SsHXK17* and *SsHXK18*/*SsHXK20*), and one tandem duplication event (*SsHXK15*/*SsHXK16*). The results showed that the expansion of SsHXKs was mainly dependent on fragment repetition events. In addition, the Ka and Ks rates and the Ka/Ks ratio of these *SsHXK* gene pairs were calculated (Table S2). All Ka/Ks ratios were <1.0 and indicated that the purifying selection was the primary pressure during the *SsHXKs* evolution period.

**Figure 4:**
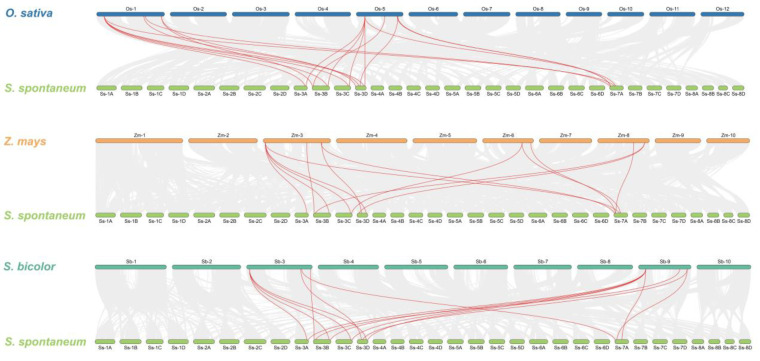
Synteny analysis of *HXK* genes between *S. spontaneum* and *O. sativa*, *S. spontaneum* and *Z. mays*, *S. spontaneum* and *S. bicolor*, respectively. Gray lines in the background indicate the collinear blocks, while the red lines highlight the syntenic *HXK* gene pairs. The chromosome number is shown at the top or bottom of each chromosome.

To investigate the homology of HXK family genes among monocots, we conducted three collinear maps between *S. spontaneum* and *O. sativa* (17 gene pairs), *S. spontaneum* and *Zea mays* (14 gene pairs), *S. spontaneum* and *Sorghum bicolor* (15 gene pairs), respectively (Figure 4). Collinear gene pairs were found only on chromosomes 3A, 3B, 3C, 3D and 7A, but not on chromosomes 7B and 7D. Collinear maps showed a high homology of *HXK* family genes among monocots.

### 2.3. Conserved Motifs and Gene Structures of the HXK Gene Family

**Figure 5:**
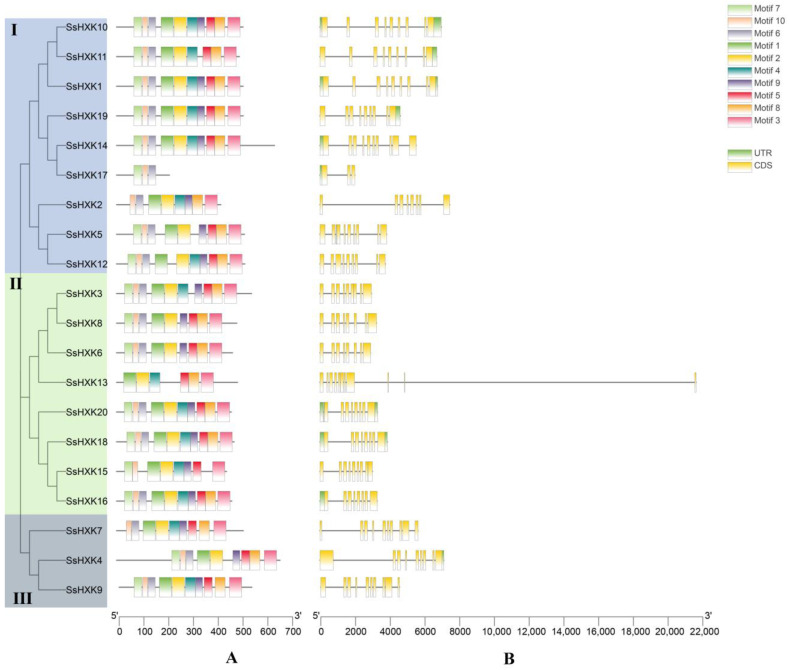
Phylogenetic tree, conserved motif, and the gene structure of the *HXK* genes. The phylogenetic tree was constructed using MEGA X software based on HXK protein sequences: (**A**) conserved motifs of the HXK proteins. Each motif is represented with a specific color; and (**B**) gene structure of *HXK* gene. The untranslated 5′- and 3′-regions, introns and exons are represented with green boxes, yellow boxes, and black lines, respectively.

To analyze the differences of protein sequences, we detected the conserved motifs using the MEME program (Figure 5). Ten motifs were identified, and the motif length ranged from 21 to 50 aa (Table S3). All *SsHXKs* had motif 10 except *SsHXK13*. Motif 3 and motif 1 were found in all sequences except the *SsHXK17*. Most SsHXK members contain eight conserved motifs. In addition, we constructed an unrooted phylogenetic tree based on SsHXK protein sequences, suggesting that the motifs classification of *HXK* genes was consistent with the phylogenetic tree.

In addition, to better understand the gene structure of the *SsHXK* genes, exons–introns were analyzed by assessing the annotation information of the GFF files in the *S. spontaneum* genome through GSDS2.0. The results showed that all *SsHXKs* contained introns, four *SsHXKs* included both 5′- and 3′-UTRs (*SsHXK1*, *SsHXK10*, *SsHXK18* and *SsHXK20*), three *SsHXKs* only contained 3′-UTR (*SsHXK14*, *SsHXK16* and *SsHXK17*), three *SsHXKs* only 5′-UTR (*SsHXK4*, *SsHXK11* and *SsHXK19*), while the remaining ten genes had no UTR region. In addition, most of the *SsHXK* genes contained 8–11 exons except *SsHXK17* (three exons).

### 2.4. CREs Analysis of SsHXK Genes

**Figure 6:**
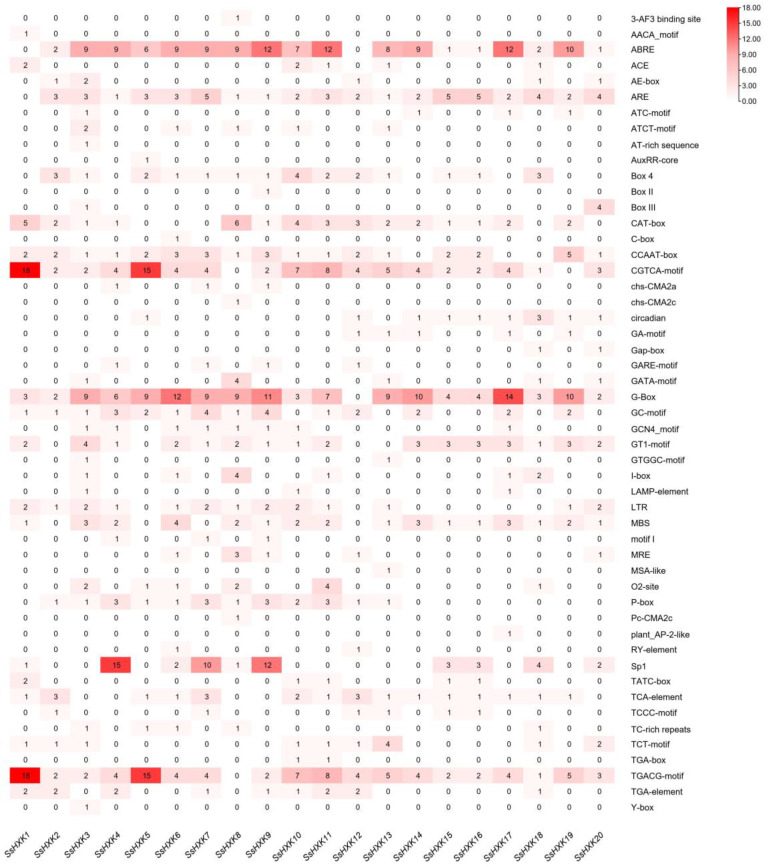
Types of cis-elements in the promoters of *SsHXK genes*. The rightmost column represents the types of cis-elements in the promoters of *SsHXK* genes. The numbers in the box are the number of cis-elements.

Putative cis-elements in the promoter regions (upstream 3000 bp) were identified using PlantCARE (results shown in Table S4). The results showed that a large number of CREs were widely distributed in the promoter region of *SsHXK* genes, such as stress responsiveness, phytohormone responsiveness, light responsiveness and growth and development (Figure 6). In the promoter regions of *SsHXKs*, the light, ABA (ABRE) and MeJA (TGACG-motif and CGTCA-motif) responsive elements were the most numerous. Among them, light response elements were identified in all members. MeJA response elements were identified in all *SsHXKs*’ promoters except the *SsHXK8*. ABA response elements were identified in all 20 members except the *SsHXK1* and *SsHXK12*. Moreover, drought responsive elements (MBS) were identified in 16 members (*SsHXK1*, *SsHXK3*, *SsHXK4*, *SsHXK6*, *SsHXK8*, *SsHXK9*, *SsHXK10*, *SsHXK11*, *SsHXK13*, *SsHXK14*, *SsHXK15*, *SsHXK16*, *SsHXK17*, *SsHXK18*, *SsHXK19*, *SsHXK20*). Low-temperature response elements (LTR) were identified in 13 members (*SsHXK1*, *SsHXK2*, *SsHXK3*, *SsHXK4*, *SsHXK6*, *SsHXK7*, *SsHXK8*, *SsHXK9*, *SsHXK10*, *SsHXK11*, *SsHXK13*, *SsHXK19*, *SsHXK20*).

### 2.5. Expression Pattern of SsHXK Genes during Saccharum Spontaneum Development

**Figure 7:**
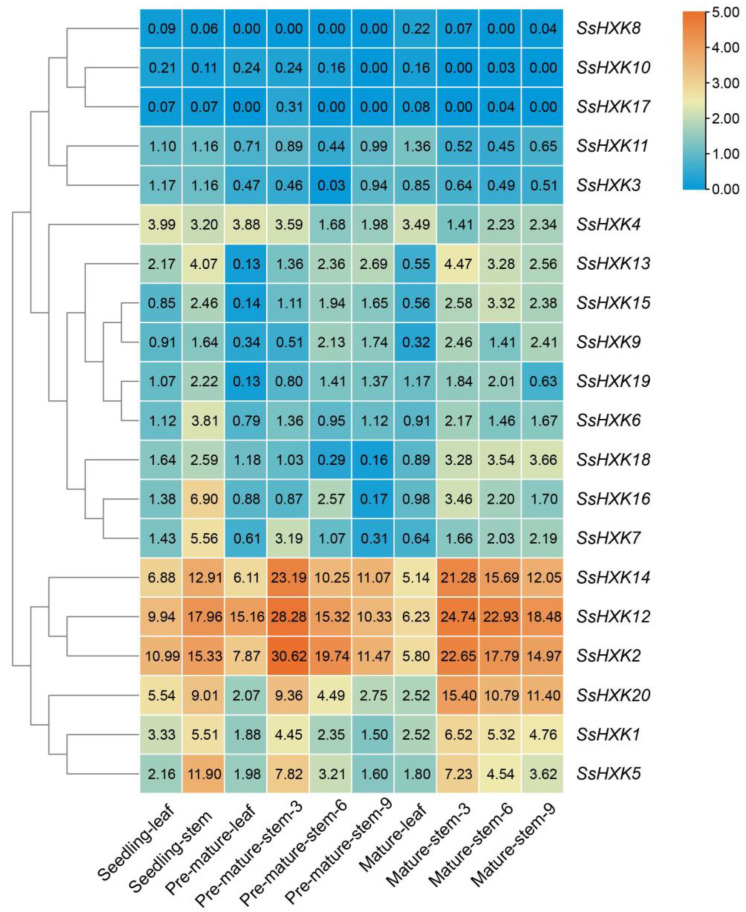
Expression profiles of the *HXK* genes from *S. spontaneum* at different development stages. The number in the box represent the origin FPKM values. Seedling, Pre-mature and Mature represented 35-day-old, 9-month-old and 12-month-old *S. spontaneum*, respectively. Stem-3, stem-6, stem-9 means the thirdly, sixth and ninth nodal stem of sugarcane from the top to bottom, respectively. The color bar represented the normalized values (log_2_ FPKM).

To understand spatiotemporal expression patterns of *SsHXKs*, we analyzed the transcriptomes of various tissues (Seedling-leaf, Seedling-stem, Pre-mature-leaf, Pre-mature-stem-3, Pre-mature-stem-6, Pre-mature-stem-9, Mature-leaf, Mature-stem-3, Mature-stem-6, Mature-stem-9) (Figure 7). In general, most of *SsHXKs* are expressed during growth and development but with a different expression. According to the expression profiles, there are 17 *SsHXKs* expressed in all ten tissues (FPKM > 0), except for three *SsHXK* genes (*SsHXK8*, *SsHXK10* and *SsHXK17*). At the same time, there were seven *SsHXK* genes (*SsHXK1*, *SsHXK2*, *SsHXK4*, *SsHXK5*, *SsHXK12*, *SsHXK14* and *SsHXK20*) whose FPKM values were greater than 1 in all tissues, indicated that these genes showed constitutive expression. Most of these *SsHXK* genes were found expressed in more than one detected organ. Three genes, *SsHXK2*, *SsHXK12* and *SsHXK14*, had similar expression patterns and were highly expressed in all tissues at different development stages, which indicated that these three genes maybe the key function genes in *S. spontaneum*. *SsHXK2*, *SsHXK12* and *SsHXK14* showed the highest expression levels in the pre-mature-stem-3 of *S. spontaneum*. Obviously, among *SsHXK1*, *SsHXK2*, *SsHXK5*, *SsHXK12*, *SsHXK14* and *SsHXK20* genes, the expression level in the stems were higher than in the leaves, which suggested that these six genes may play a more important role in the growth and development of stem. What’s more, these six genes showed a decline from the top (pre-mature-stem-3) to bottom (pre-mature-stem-9) of the stem during sugar accumulation stage, which suggested that these six genes may be involved in the process of sugar metabolism in sugarcane stems.

### 2.6. Expression Pattern of SsHXK Genes under Cold and Drought Treatments

**Figure 8:**
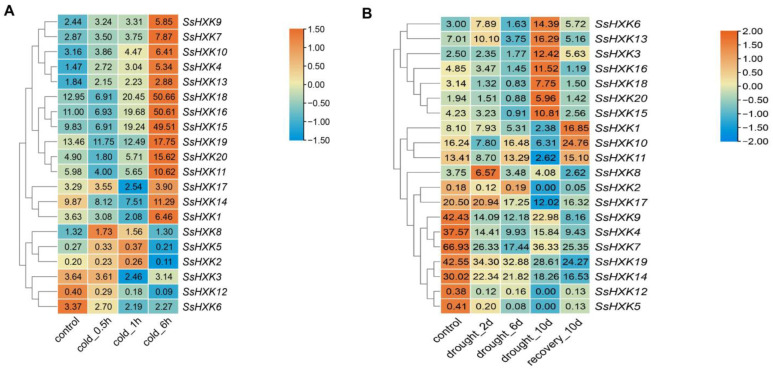
Expression profiles of *SsHXKs* based on SRA data. (**A**) Sugarcane stressed by cold. (**B**) Sugarcane stressed by drought. The number shown in the box is the original trimmed mean of M-values (TMM) which represent the expression levels of *SsHXKs*. The color bar represented the row scale normalized values.

*SsHXK* genes contained numerous cis-elements responding to drought and cold stress in the promoter regions, so we analyzed the expression patterns of *SsHXKs* under drought and cold stress to further investigate the potential functions of *SsHXK* genes (Figure 8). Under cold stress, the *SsHXKs* had different expression profiles and were expressed at all time points. After cold treatment for six hours, the expression level of 14/20 *SsHXKs* was significantly higher than that of control group and other treatment groups, especially *SsHXK18*, *SsHXK16* and *SsHXK15*. While *SsHXK2*, *SsHXK3*, *SsHXK5* and *SsHXK8* had the relatively stable expression levels after cold for six hours. The expression level of *SsHXK6* and *SsHXK12* genes had a continuous downward trend after cold treatment.

As for drought treatment, all genes were expressed except for *SsHXK2*, *SsHXK5* and *SsHXK12*. Especially, these three genes were not expressed after ten days of drought treatment. After drought treatment for ten days, the expression level of seven *SsHXKs* (*SsHXK3*, *SsHXK6*, *SsHXK13*, *SsHXK15*, *SsHXK16*, *SsHXK18* and *SsHXK20*) was higher than that other groups. After ten days of recovery, three genes (*SsHKX1*, *SsHKX10* and *SsHKX11*) had a higher expression level than the control group and all of drought treatment groups. Moreover, seven *SsHXKs* (*SsHXK4*, *SsHXK5*, *SsHXK7*, *SsHXK9*, *SsHXK12*, *SsHXK14* and *SsHXK19*) were found that the expression profiles had a continuous downward trend. These results showed that *SsHXKs* may play different roles in response to drought and cold stress.

## 3. Discussion

HXKs family proteins play a key regulatory role in sugar sensing, sucrose metabolism, catalyzing hexose phosphorylation, providing energy and modulation of plant growth and stress adaptation [27]. HXK belongs to a multigene family and HXK is usually encoded by a medium family of approximately 4–10 genes in higher plant, i.e., six members of the HXK family were identified in *A. thaliana* [2], while it is represented by 10 members in *O. sativa* [28], nine members in *Z. mays* [29], nine members in *Nicotiana tabacum* [30], 17 members in *Gossypium hirsutum* L. [31] and 19 putative members in *B. napus* [32] (Table S5).

However, little information about the *HXK* gene family has been reported in sugarcane. The assembled genome of *S. spontaneum* AP85–441 served as convenient reference for analyzing the *SsHXK* gene family in sugarcane. In the present study, 20 *SsHXK* genes were identified from the whole genome of *S. spontaneum* AP85–441, which was more than that in *O. sativa* (10 *HXKs*) [28], *Z. mays* (9 *HXKs*) [29] and *A. thaliana* (6 *HXKs*) [2]. Is the number of *HXKs* gene family related to the genome size of the species? To answer this question, we compared the size of the selected plant species’ genomes and the number of HXK family genes, i.e., *O. sativa* (466 Mb/10 *HXKs*), *A. thaliana* (125 Mb/6 *HXKs*), *Z. mays* (2.3 Gb/9 *HXKs*), and *S. spontaneum* (3.36 Gb/20 *HXKs*). The results showed that the number of members of the *HXK* gene family was not positively correlated with the size of the plant genome. The number of *HXK* genes varied widely among different plant species, suggesting that the *HXKs* were not conserved during the evolution progress. Moreover, the *S. spontaneum* AP85–441 was haploid, produced by the octoploid SES208, considering the expansion of gene families in polyploidy, the number of *HXK* genes in octoploid *S. spontaneum* most likely could be over 20.

Sugarcane is an allopolyploid and aneuploid perennial herbaceous crop with 100~120 chromosomes [26,33]. Zhang et al. have speculated that the sugarcane genome undergone whole-genome duplication, random duplication and segmental duplication, and during intergeneric hybridization or domestication [34].In the present study, 20 HXK members of sugarcane are located on seven of 32 chromosomes(Chr3A, Chr3B, Chr3C, Chr3D, Chr7A, Chr7B and Chr7D), while nine *ZmHXKs* of *Z. mays* [29], six *AtHXKs* of *A. thaliana* [2], and ten *OsHXKs* of *O. sativa* [28] are all located on three of their own chromosomes. *ZmHXKs* were located on chromosomes 3, 6 and 8 [29], *AtHXKs* were located on chromosomes 1, 5 and 7 [2], while *OsHXKs* were located on chromosomes 1, 5 and 7 [28]. The present study showed that the chromosome distribution of *SsHXKs* was uneven. For example, the chromosome Chr3D contained five *SsHXK* genes, while the chromosome Chr3A and Chr3B both have three *SsHXK* genes, respectively. Similar results were also obtained for the chromosome Chr7A, Chr7B and Chr7D. These findings indicated that whole-genome duplication and segmental duplication might have occurred on these seven chromosomes, which was consistent with the findings of Zhu et al. [3].

In addition, phylogenetic analysis of the *HXKs* among *S. spontaneum*, *A. thaliana* and *O. sativa* resulted in their clustering into four subgroups, named group I, group II, group III and other. *AtHKL3* is a single branch, which is similar to the phenomenon of *ZmHXK* phylogenetic tree construction [29]. Moreover, according to the present evolutionary tree branch and HXK studies by Cho et al. and Zheng et al. [28,35], we speculated that SsHXK4, SsHXK7 and SsHXK9 are HKL proteins, and the rest are HXK proteins.

To determine the evolution of the *SsHXK* genes, gene structures and conserved motifs were conducted. The gene structure analysis suggested that most of *SsHXK* genes were found 8–11 exons, which is similar to those in *Z. mays* [29], *G. hirsutum* L. [31] and *B. napus* [32]. Conserved motif analysis showed that all SsHXK members had typically conserved region, which is consistent with that in other species, i.e., *A. thaliana* [2], *Z. mays* [29], *G. hirsutum* L. [31] and *B. napus* [32]. These findings indicated that the *SsHXK* genes in sugarcane were extremely conserved during the evolutionary process, intergeneric hybridization or domestication.

Cis-acting elements in promoters play a very important role in regulating phytohormone and stress response. We found that the SsHXK family members contain numerous elements related to phytohormone response, light response, and stress responses. All 20 *SsHXKs* contained ABA response elements except *SsHXK1* and *SsHXK12*, which suggesting that most *SsHXKs* may regulate phytohormone sensitivity, which is consistent with the findings by Moore et al. that overexpression of *AtHXK1* in *A. thaliana* can result in increased cytokinin sensitivity [7].

Segmental or tandem duplicates in many gene families are the main expanding pattern in plants [36]. We detected the *SsHXK* genes duplication events, and 11 *HXK* gene pairs were identified as fragment duplicates, while one tandem duplication event (*SsHXK15*/*SsHXK16*). These results clearly showed that the expansion of *SsHXKs* in sugarcane genome was mainly dependent on fragment repetition events, which is consistent with the results of a previous study [35]. Similar phenomenon of low tandem and high segmental duplication proportion for gene families were also found in *BrVQ* and *ZmVQ* gene family [37,38]. Ka/Ks ratio of *SsHXks* results indicated that the purifying selection were the primary pressure during the *SsHXKs* evolution period. Collinear maps of between *S. spontaneum* and *O. sativa*, *S. spontaneum* and *Z. mays*, *S. spontaneum* and *S. bicolor*, showed high homology of HXK family genes among monocots.

Tissue-specific expression patterns could provide a better understanding of gene function in plants [3,39]. In our study, the *SsHXK* genes showed diverse expression patterns in ten different tissues. Of the twenty *SsHXK* genes, seventeen genes were expressed in all the ten evaluated tissues, while three genes, *SsHXK8*, *SsHXK10* and *SsHXK17* were detected almost no expression in any evaluated tissues, which suggested that the expression of *SsHXK* genes is selective. The present study found that six genes (*SsHXK1*, *SsHXK2*, *SsHXK5*, *SsHXK12*, *SsHXK14* and *SsHXK20*) were higher expressed in the stems than in the leaves, indicating that these genes may be more involved in stem metabolism and basic cellular activities, especially in the progress which may be related to sugar storage in stems of sugarcane [3]. A weak decrease of these six genes’ expression was found from the top to bottom of the stem during the sugar accumulation stage, of which the same expression patterns of *SsWRKYs* were reported by Li et al. [40].

Expression profiles displayed by transcriptome data can provide a global perspective to examine gene family expression [26,40]. SRA data of transcriptomes of ten specific tissues of sugarcane ROC22 were employed to investigate the role of *SsHXKs* gene in response to low temperature and drought. The *SsHXK* expression levels from SRA showed that most *SsHXKs* were up-regulated or down-regulated, which is consistent with the prediction that the promoter region contained a large number of ABRE, ARE, G-Box, LTR and MBS response elements, which are important components in response to abiotic stresses such as drought, low temperature through ABA pathway or others [41,42]. The expression level of *SsHXK15*, *SsHXK16* and *SsHXK18* were higher than other groups after cold for 6 h. On the contrary, *SsHXK4* and *SsHXK9* had lower expression level than control. As for drought treatment, three *SsHXKs* (*SsHKX1*, *SsHKX10* and *SsHKX11*) had the highest expression level after 10 days of recovery. Overall, our results primarily revealed the potential biological function of *SsHXKs*, which may provide information for in depth functional verification.

## 4. Materials and Methods

### 4.1. Identification of HXK Gene Family in Sugarcane

The genomic information of sugarcane was come from the *S. spontaneum* AP85–441 genome (http://www.life.illinois.edu/ming/downloads/Spontaneumgenome/, accessed on 5 May 2022) [34]. HXK domains (Pfam number PF00349 and PF03727) were used to search against *S. spontaneum* AP85–441 genome using HMMER 3.0 with the values (e-value) cut-off at 0.1. The candidate *SsHXKs* were submitted to SMART (http://smart.emblheidelberg.de/, accessed on 6 May 2022) and Conserved Domain Database (https://www.ncbi.nlm.nih.gov/Structure/cdd/wrpsb.cgi, accessed on 6 May 2022) to confirm that they all contained the HXK domain and manually remove members that do not have HXK domain(Figures S1 and S2). Physical parameters of HXK proteins, including protein length, isoelectric point (pI), and molecular weight were calculated in ExPASy (http://www.expasy.org/tools, accessed on 14 May 2022). Subcellular localization was predicted using the WoLF PSORT (http://wolfpsort.org/, accessed on 14 May 2022).

### 4.2. Phylogenetic Analysis and Gene Duplication Analysis of SsHXK Proteins

The neighbor-joining (N-J) phylogenetic tree among *A. thaliana*, *O. sativa* and *S. spontaneum* was constructed using MEGA X software with 1000 bootstrap replications [43]. AtHXK and OsHXK proteins were downloaded from phytozomes (http://www.phytozome.org, accessed on 20 May 2022). The chromosomal distribution of the identified *SsHXK* genes was obtained and visualized using TBtools software (v.1.0971) [44]. The gene duplication events were conducted by using MCScanX [45]. Non-synonymous (ka) and synonymous (ks) substitutions of each duplicated *SsHXK* genes were calculated using KaKs Calculator 2.0 [46].

### 4.3. Gene Structure and Motif Composition of SsHXK Genes

Conserved motifs of HXK proteins were identified using MEME (v.5.3.3) (http://meme-suite.org/tools/meme/, accessed on 24 July 2022) with default settings [47]. Gene structure was investigated using GSDS 2.0 (http://gsds.cbi.pku.edu.cn/, accessed on 25 July 2022) [48]. TBtools was used to integrate phylogenetic tree, gene structure results, and conserved motifs.

### 4.4. Promoter Analysis of SsHXK Genes

The 3000 bp sequences upstream of the *SsHXK* genes transcriptional start site were submitted to PlantCARE (http://bioinformatics.psb.ugent.be/webtools/plantcare/html/, accessed on 10 August 2022) to identify the putative cis-elements [49].

### 4.5. Expression Analysis of SsHXKs Based on RNA-Seq

Firstly, sugarcane different tissue expression data were download from *Saccharum* Genome database (http://sugarcane.zhangjisenlab.cn/sgd/html/index.html, accessed on 10 August 2022). In addition, the public data (PRJNA590595 and PRJNA636260) were download from the SRA database about drought and cold stress in ROC22. There are ten transcriptomes of tissues from Seedling-leaf, Seedling-stem, Pre-mature-leaf, Pre-mature-stem-3, Pre-mature-stem-6, Pre-mature-stem-9, Mature-leaf, Mature-stem-3, Mature-stem-6, Mature-stem-9 (Seedling, Pre-mature and Mature represented 35-day-old, 9-month-old and 12-month-old *S. spontaneum*, respectively. Stem-3, stem-6, stem-9 means the thirdly, sixth and ninth nodal stem of sugarcane from the top to bottom, respectively). Then, Fastq and hisat2 tools were applied to get clean reads and map sequence data on the reference genome (*S. spontaneum* AP85–441), respectively [50,51]. The featurCounts in Subread package and trimmed mean of M-values (TMMs) were conducted for count read and normalization. Finally, all the expression values (FPKMs or TMMs) were used to make heat maps and cluster analysis by TBtools.

## 5. Conclusions

In the present study, 20 *SsHXK* genes were identified from the *S. spontaneum* genome and were classified into four groups. Most *SsHXKs* contained 8–11 introns which was consistent with other monocots. Duplication event analysis indicated that *HXKs* in *S. spontaneum* L. primarily originated from segmental duplication. Promoter analysis and expression analysis suggested that *SsHXK* genes may play an important role in the process of development and response to abiotic stress. These results provide valuable resources to better understand the biological role of the sugarcane *HXK* genes.

## Data Availability

The datasets generated for this study can be found in SRA, the accession number: PRJNA590595 and PRJNA636260.

## Supplementary Materials

The following supporting information can be downloaded at: https://www.mdpi.com/article/10.3390/plants12061215/s1, Table S1: The detailed information of *HXK* genes included in this study; Table S2: Syntenic relationships of *HXK* genes in *Saccharum spontaneum*; Table S3: The detail information of HXK conserved motif; Table S4: Promoter cis-element analysis of the *HXK* genes from *Saccharum spontaneum*; Table S5: The HXK gene family from different species; Figure S1: the domain of the SsHXKs family; Figure S2: Amino acid sequence alignment of the glucose-binding domain in *Arabidopsis thaliana* (At), AtHXK1, AtHXK2, and SsHXKs.
