# Supplementary figures and images for: Identification and Expression Analysis of Hexokinases Family in Saccharum spontaneum L. under Drought and Cold Stresses
Source: Plants (Basel). 2023 Mar 7;12(6):1215. doi: 10.3390/plants12061215 (PMC10056587; doi:10.3390/plants12061215)

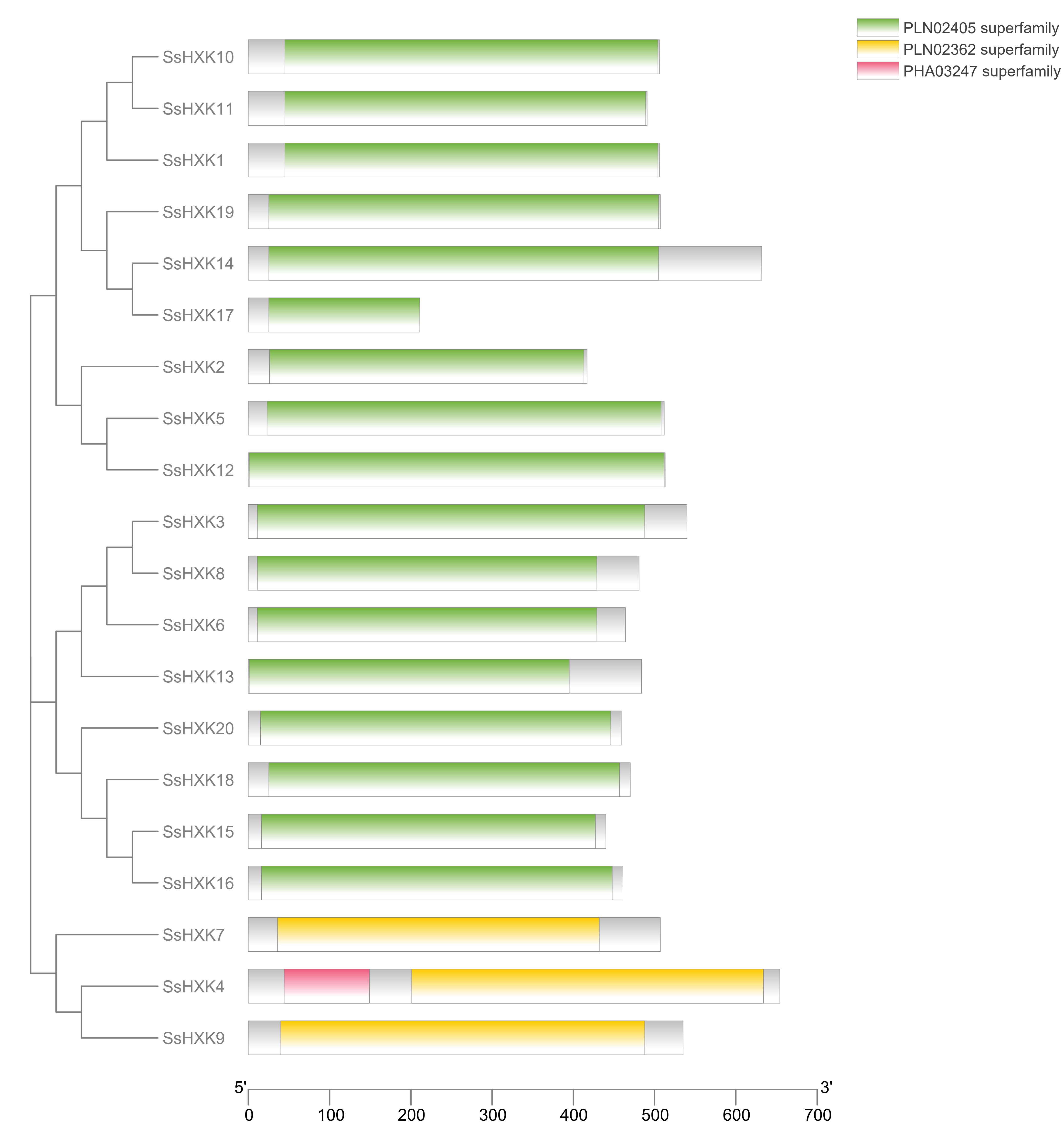

Supplement: Supplementary file 1 [file plants-12-01215-s001.zip › Figure S1 HXK-domain.jpg]

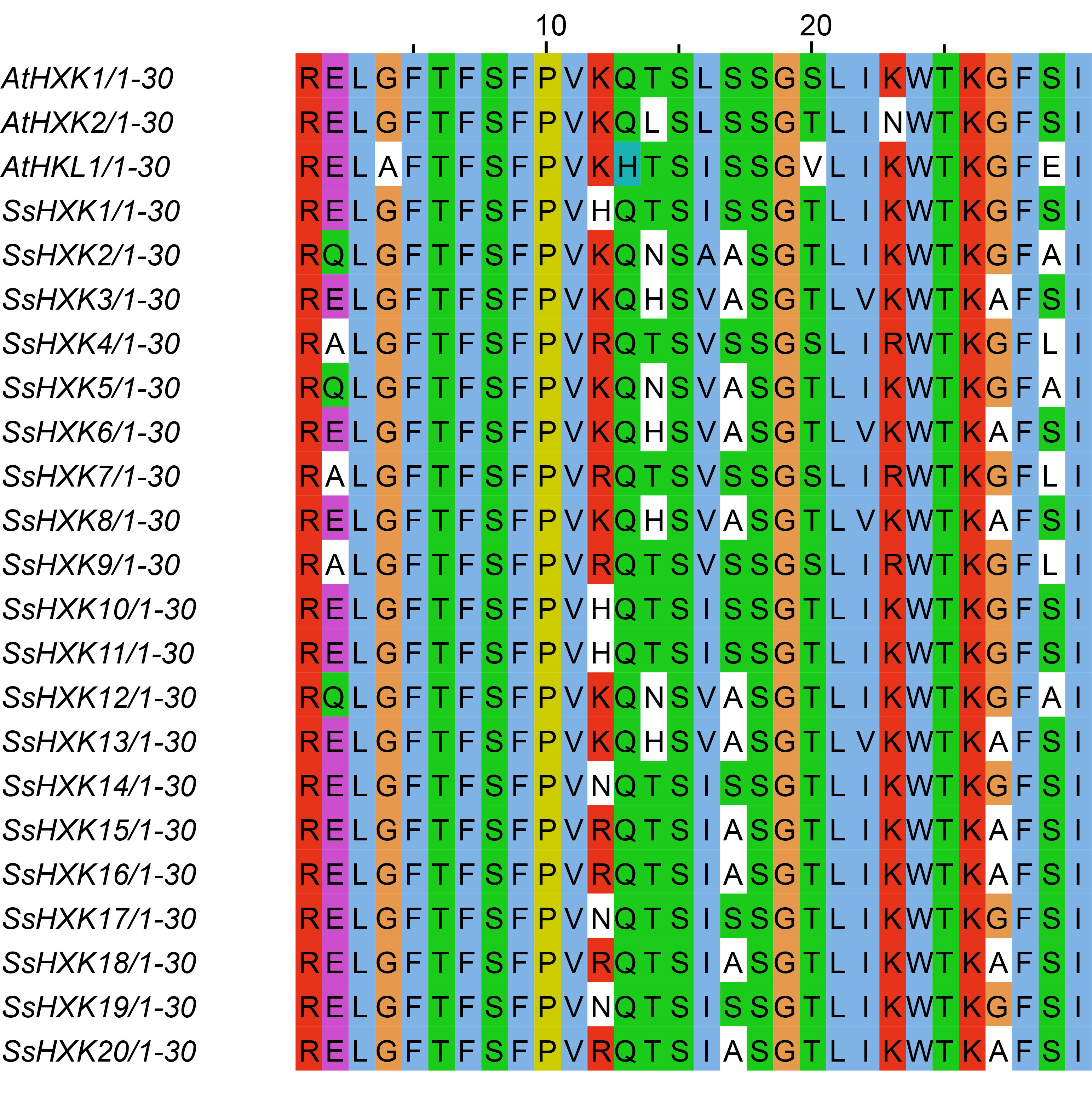

Supplement: Supplementary file 1 [file plants-12-01215-s001.zip › Figure S2 multiple sequence alignment.jpg]
